# Optimizing the Manufacturing Process Control of Si-Based Soft Magnetic Composites

**DOI:** 10.3390/ma18102321

**Published:** 2025-05-16

**Authors:** Seongsu Kang, Seonbong Lee

**Affiliations:** 1 Department of Mechanical Engineering, Keimyung University, Daegu 42601, Republic of Korea; 5472068@stu.kmu.ac.kr; 2 Department of Automotive Engineering, Keimyung University, Daegu 42601, Republic of Korea

**Keywords:** motor, soft magnetic composites (SMC), powder metallurgy (PM), microstructure, morphology

## Abstract

This study attempts to enhance the formability and electromagnetic properties of Fe-Si-based soft magnetic composites via process parameter optimization. Two silicon compositions (5.0 and 6.5 wt.%) were examined to determine their influence on density, internal stress, microstructure stability, and magnetic properties using a factorial design comprising 96 different condition combinations. A Pearson correlation analysis revealed a negative relationship between Si content and formability, while magnetic permeability increased with higher Si content. The 5.0 wt.% Si samples exhibited superior density (7.42 g/cm^3^ vs. 7.28 g/cm^3^), uniform microstructure, and coating stability. Conversely, the 6.5 wt.% Si samples achieved better permeability (126 at 10 kHz) than 5.0 wt.% Si samples but exhibited higher internal stress, uneven compaction, and thicker insulation layers (~400 nm vs. <10 nm). Scanning electron microscopy and transmission electron microscopy analyses identified necking and damage to the insulation layer. X-ray diffraction verified the stability of the Fe_1.6_Si_0.4_ phase after the forming and annealing processes. Secondary molding temperature exhibited the most significant impact on densification, and annealing generally degraded the quality factor (Q-factor). The highest Q-factor value (7.18 at 10 kHz), indicating lower core loss, was observed in the 5.0 wt.% Si samples without annealing.

## 1. Introduction

The recent electrification trend in the green automotive industry and increasing demand for high-efficiency, low-cost industrial applications have driven a growing need for energy- and cost-efficient electromagnetic core components. In the conventional automotive market, motors are dominated by rotors and stators that employ laminated electrical steel plates, which are prevalent as core components in motors due to their relatively simple and economical manufacturing process and stable magnetic properties. However, laminated electrical steel plates are limited by the abrupt increase in core loss as frequency increases and manufacturing components encounter difficulties with complex geometries. Motors in electrified eco-friendly vehicles require higher efficiency and precision, which has resulted in the growing attention on alternative materials and processes that can address the limitations of conventional laminated electrical steel plates. Accordingly, soft magnetic composites (SMCs) are emerging as next-generation materials capable of replacing laminated electrical steel plates [1,2,3,4,5,6].

SMCs are formed by coating iron powder particles with an insulating layer, which provides them with several key advantages over conventional laminated electrical steel plates. First, SMCs exhibit isotropic magnetic properties, enabling three-dimensional magnetic circuit designs and the manufacturing of components with complex geometries, which is not readily achievable with laminated electrical steel plates. In addition, their high electrical resistivity results in reduced eddy current losses, rendering them suitable for high-frequency applications. They also exhibit lower core loss than laminated electrical steel plates, offering significant advantages in high-speed traction motors for electric vehicles. Mechanically, SMCs maintain a uniform microstructure and high strength while enabling greater design flexibility in component shapes. Furthermore, their powder compaction manufacturing process, rather than conventional machining, minimizes material waste and enhances production efficiency, rendering them highly economical [7,8].

Fe-Si alloy powder is particularly suitable for motor core applications among SMC materials due to its remarkable magnetic and electrical properties. The primary advantage of Fe-Si material is its high electrical resistivity, which effectively suppresses eddy current losses. Compared to pure Fe-based SMCs, introducing Si increases electrical resistivity, thereby reducing magnetic losses at high frequencies and enhancing performance in high-speed rotating motors and high-frequency applications. Regarding practical application, controlling the Si content in Fe-Si-based SMCs is critical, influencing both electromagnetic performance and mechanical formability. Various silicon compositions, typically ranging from 1.5 wt.% to 6.5 wt.%, have been investigated. Although increased Si content improves electrical resistivity and enhances magnetic properties such as permeability, excessive Si addition increases brittleness, reducing the powder’s plastic deformability and flowability, thus degrading formability and increasing the risk of internal defects during compaction. To address this fundamental trade-off, selecting an optimized Si content that balances electromagnetic efficiency with processability is crucial. Here, Fe-Si powders containing 5.0 wt.% and 6.5 wt.% silicon were selected to examine the trade-off between formability and magnetic properties. Toroidal cores were fabricated under varying compaction and heat treatment conditions, and their density and electromagnetic performance were compared via experimental analysis [9,10,11,12,13,14,15].

The two-press two-curing (2P2C) process comprises primary compaction, annealing, cooling, secondary compaction, and a final heat treatment to yield a high-density product with excellent mechanical and magnetic properties. Primary compaction densifies the initial powder into a basic shape, either at room temperature or using a warm die at 500 °C, where high density can be achieved even under relatively low pressure. The first annealing process promotes initial particle bonding, prevents oxidation, and ensures intermediate mechanical strength while stabilizing the microstructure, relieving residual stress, and preparing the material for secondary compaction. Secondary compaction further densifies the material by discarding residual pores from the primary stage, and because it is conducted under warm die conditions, plastic deformation is facilitated. In the final stage, the second heat treatment completes the particle bonding and forms a uniform microstructure, optimizing mechanical strength and magnetic properties [16,17,18].

Powder metallurgy (PM) processes using SMCs powders theoretically offer high design flexibility and the ability to form complex three-dimensional structures. However, achieving uniform compacts with maintained high magnetic performance is challenging in practical manufacturing. This issue is more pronounced in large or complex-shaped components, such as traction motor parts for electric vehicles, where the increased cross-sectional area during forming necessitates high compaction pressure. Consequently, this results in various productivity issues, including the need for advanced equipment specifications, reduced forming die durability, and increased defect rates [19]. Moreover, the inherently low flowability of SMC powders often results in uneven powder filling during the forming of complex geometries, thus increasing internal porosity in the final product and directly degrading in both mechanical and magnetic properties [20].

To address such process-related limitations, several studies have been conducted on the forming conditions of SMCs. XU, Weijie et al. extended the conventional Stoner–Wohlfarth (S–W) model by incorporating stress-dependent magnetization behavior, considering the influence of internal residual stresses induced during compaction on the magnetic hysteresis of SMCs. Their model demonstrated that compressive and tensile stress states can alter the preferred magnetization direction, thus modulating hysteresis loss. The model was experimentally validated via stress-controlled magnetic measurements, providing strong evidence for the correlation between mechanical stress and core loss behavior [21]. Meanwhile, Huang, Haohui et al. enhanced both the compact density and magnetic properties of FeSiCr-based SMCs using warm compact techniques. Their findings indicated that the application of an appropriate forming temperature diminishes the resistance to magnetic domain rotation, thus enhancing permeability and minimizing core loss [22]. These approaches, which involve precise control of powder characteristics and forming conditions, are being presented as practical alternatives for improving the performance of SMCs. Employing polytetrafluoroethylene (PTFE) as an insulating coating material to fabricate iron-based SMCs, Song et al. systematically investigated the effects of PTFE content, compaction pressure, and annealing treatment on magnetic properties. Under optimal conditions—3 wt.% PTFE content, 648 MPa compaction pressure, and annealing at 150 °C for 90 min—the specimens exhibited excellent performance, including an effective permeability of 56, saturation magnetization of 192.9 emu/g, and total core losses of 355 mW/cm^3^ (50 mT, 50 kHz) and 1705 mW/cm^3^ (100 mT, 50 kHz). This study underscores PTFE’s potential as a viable insulation material and provides a comprehensive understanding of the correlation between processing parameters and magnetic performance, offering practical insights for developing high-efficiency iron-based SMCs [23].

Fe-Si-based materials have emerged as foremost promising candidates for high-speed and high-frequency applications among various SMC systems due to their relatively high electrical resistivity, low eddy current losses, and stable magnetic behavior. These material advantages have led to increasing interest in the precise control of forming parameters, insulation chemistry, and thermal treatment to further enhance performance.

Despite recent progress, systematic and integrated studies simultaneously addressing formability, microstructural stability, and electromagnetic performance remain limited. Hence, this study fabricated toroidal cores using Fe-Si powders with varying silicon contents and quantitatively analyzed the effects of key process parameters on the final density and magnetic properties. Accordingly, practical process guidelines are proposed to improve both the performance and manufacturability of Fe-Si-based SMCs.

The study was conducted in the following steps:

1. The selection of Fe-Si-based SMC powders with varying Si contents and formulation of an experimental design considering major influencing process parameters.
2. A microstructural analysis and property evaluation of the Fe-Si powders.
3. Experimental fabrication of toroidal cores according to the established design, followed by an analysis of the effects of each major parameter on the magnetic and physical characteristics of resultant compacts.
4. The identification of dominant factors using Pearson correlation coefficient (PCC) analysis.
5. A simulation-based analysis to assess internal stress and density deviation, with subsequent verification of the simulation reliability via a comparison with experimental data.

The aforementioned process enables a comparative evaluation of the two Si contents’ performance, including the derivation of guidelines for assessing the properties of prototypes.

## 2. Materials and Methods

Fe-Si-based SMCs have emerged as the next generation core materials for high-performance and high-efficiency motors. Compared to conventional laminated electrical steel plates, Fe-Si materials exhibit superior, excellent electromagnetic properties and reduced iron losses due to their high electrical resistivity, rendering them suitable for high-speed electric motors and xEV motor applications [24]. Here, the Fe-Si content was adopted to balance electromagnetic performance and mechanical strength. Based on prior research, Si contents of 5.0 wt.% and 6.5 wt.% were selected for application, and the effects of each composition on formability and electromagnetic properties were evaluated.

The generally accepted optimum content for electromagnetic properties is 6.5 wt.%. High Si content enhances electrical resistivity, effectively suppresses eddy current losses, and provides optimal performance relative to permeability and saturated flux density. However, at 6.5 wt.%, the high brittleness of silicon complicates molding, and the high pressing force increases the risk of internal cracking. In addition, high compression molding can result in poor inter-powder cohesion, increasing internal porosity and degrading insulation performance after sintering and heat treatment. To address these challenges, a strategy was adopted to secure formability and mechanical strength by considering a 5.0 wt.% Si composition with reduced Si content. Consequently, two compositions, 5.0 wt.% and 6.5 wt.%, were compared and analyzed to ensure a balance between mechanical performance, formability, and electromagnetic performance in Fe-Si materials.

### 2.1. Fe-Si Material

Here, powder characterization, including morphology and composition analyses, was conducted to determine the suitability of the Fe-Si material. Accordingly, the physical and chemical properties of Fe-Si powders were quantitatively analyzed, and their internal formability and thermal properties were examined for their suitability as SMC cores.

#### 2.1.1. Mechanical Properties of Fe-Si

**Figure 1:**
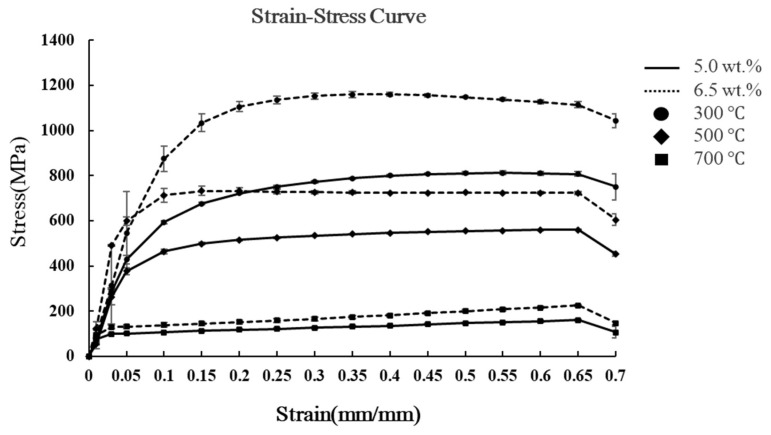
Strain–stress curves of Fe-Si powder.

To evaluate the formability of Fe-5.0 wt.% Si powder, high-temperature compression tests were performed to derive strain–stress curves, adhering to ASTM E209 test specifications and utilizing a Gleeble dynamic thermometer tester (Gleeble 3800-GTC, Gleeble, Poestenkill, NY, USA) as the test equipment [25]. The measuring temperatures were set at 300 °C, 500 °C, and 700 °C, with a fixed strain rate of 0.1 mm/s. These high-temperature compression tests involved applying compressive strain at a controlled temperature and constant pressing rate to evaluate powder deformation behavior during molding and quantitatively analyze the internal stress distribution. The strain–stress curves, derived by measuring stress changes with increasing strain, were employed to analyze plastic deformation characteristics and internal pore evolution. In addition, volume change and internal density distribution with molding pressure were evaluated to determine optimal pressing conditions. The strain–stress relationship of Fe-6.5 wt.% Si powder, as reported in previous studies, is illustrated in Figure 1 [26].

The evaluation of internal formability can be utilized to determine the uniformity of the compressive behavior of Fe-Si powder during SMC core molding and to assess the internal stress distribution and potential for achieving mechanical strength. Figure 1 illustrates the strain–stress curve of Fe-Si as a function of Si content and temperature. The results indicate that a 6.5 wt.% Si content yields higher stress than 5.0 wt.%, attributed to increased brittleness with higher Si concentration. Elevated temperatures result in lower mechanical stress results for both compositions, with minimal difference observed at 700 °C.

#### 2.1.2. Thermal Properties of Fe-Si

The thermal conductivity and coefficient of thermal expansion were measured from room temperature (25 °C) up to 900 °C. Thermal conductivity tests adhered to the KS L 1604 standard, utilizing the LFA 427 apparatus from NETZSCH (NETZSCH, Selb, Bayern, Germany) [27]. Disk-type specimens, fabricated in compliance with KS L 1604 with dimensions of 12.7 mm diameter and 2 mm height, were employed for these measurements. The measured thermal conductivity values are presented in Table 1 and Table 2. The thermal conductivity and coefficient of thermal expansion of Fe-6.5 wt.%Si, as reported in previous studies, are cited in Table 2 and Table 3 [26].

Thermal expansion coefficients were evaluated from room temperature (25 °C) to 900 °C using a DIL 402 HT dilatometer (NETZSCH, Selb, Bayern, Germany) following ASTM E228 [28]. Cylindrical specimens with a 5 mm diameter and 25 mm in height, fabricated in accordance with ASTM E228, were adopted for these measurements. The resulting data for the thermal expansion coefficients are presented in Table 3.

#### 2.1.3. Chemical Composition and Crystal Structures of Fe-Si

X-ray fluorescence analysis was conducted using an SEA 1200 VX instrument (Seiko Instruments Inc., Chiba, Japan) to investigate the chemical composition and elemental distribution of the Fe-Si raw powder and bulk prototypes. The resulting elemental analysis data are provided in Table 4 and Table 5.

X-ray diffraction (XRD) analysis was conducted to examine the crystal structure and chemical composition of the Fe-Si powders. Accordingly, the powder specimens were analyzed using an X’Pert PRO MPD diffractometer (PANalytical B.V., Almelo, The Netherlands), while the bulk specimens were examined with a SmartLab X-ray diffractometer (Rigaku Corporation, Tokyo, Japan). Comparing the XRD patterns of the powder and bulk samples allowed for the identification of potential alterations in crystal structure during high-temperature molding.

**Figure 2:**
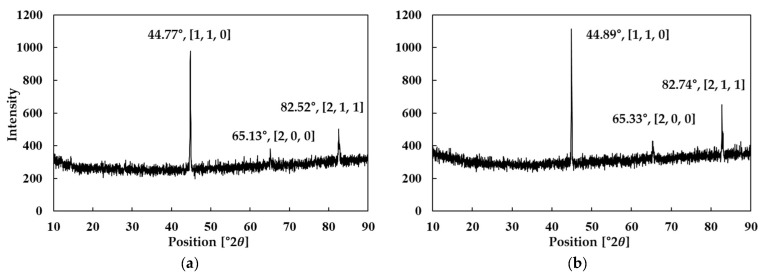
XRD results of Fe-Si powder: Si content (**a**) 5.0 wt.% and (**b**) 6.5 wt.%.

Figure 2 presents the XRD results for Fe-Si powders containing 5.0 wt.% and 6.5 wt.% Si, respectively. The analysis indicates that the primary Bragg peaks for both compositions correlated well with the Fe_1.6_Si_0.4_ crystal structure, verifying the presence of a cubic phase [29]. This cubic structure offered high magnetic stability and low magnetic anisotropy, rendering it suitable for SMC applications.

#### 2.1.4. Powder Morphology Analysis of Fe-Si

To evaluate the microstructure of the Fe-Si powder, various high-resolution analytical techniques were employed to analyze its morphology. Surface morphology and particle shape were observed using a scanning electron microscope (SEM; JEOL Ltd., Tokyo, Japan). In addition, particle size distribution was measured with a Mastersizer 3000 laser diffraction particle size analyzer (Malvern Panalytical Ltd., Worcestershire, UK).

The Fe-Si raw powder adopted in this study was prepared by a water atomization process, and the average particle size was selected to be approximately 100 μm to ensure good formability in PM and optimal magnetic properties. Generally, excessively fine powder particles hinder uniform molded body production due to interparticle agglomeration and require high compression force. In contrast, excessively large particles increase internal porosity, resulting in density loss and electromagnetic performance degradation. Therefore, based on previous research demonstrating that powders in the 75–150 μm range facilitate uniform powder filling and molding and enable optimal density and magnetic properties (low iron loss, high permeability, etc.), this study selected an average particle size of 100 μm, which was determined to be the best process condition via experimental evaluation [13,30,31,32].

**Figure 3:**
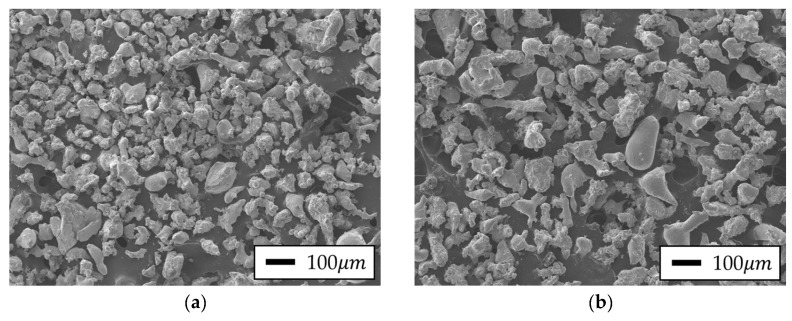
SEM results of Fe-Si Powder: Si content (**a**) 5.0 wt.% and (**b**) 6.5 wt.%.

The obtained powder morphology is illustrated in Figure 3, and the Dv 50 level results of the particle size test are presented in Table 6. Regarding 5.0 wt.% Si content, the powder was prepared with an average particle size of approximately 80 μm, and for the 5.0 wt.% silicon content, the powder was prepared with a particle size of 100 μm, considering the brittleness of silicon [31,32].

### 2.2. Experiment Methods

#### 2.2.1. Coating Conditions and Insulation Composition

Here, insulating compositions using H_3_PO_4_, polyimide (PI), and MoS_2_ were adopted to optimize the electromagnetic performance and insulating properties of Fe-Si-based SMCs. The corresponding coating compositions are presented in Table 7. The insulating coating was employed to form an insulating layer on the powder surface and minimize magnetic losses. H_3_PO_4_ forms a phosphate layer on the surface of Fe-Si powder, which increases electrical resistance and suppresses eddy current losses, and forms a stable insulating layer at high temperatures. PI is a high-temperature stable polymer insulation material with remarkable thermal and mechanical durability and high insulation properties. MoS_2_ is a layered compound with high electrical resistivity, which exhibits natural solid lubrication properties, enhances insulation properties, and reduces internal stress, contributing to improved density and reduced mold failure [33,34,35,36,37,38,39,40].

#### 2.2.2. Experimental Factor Settings

Silicon content, molding temperature, and annealing treatment were identified as key factors significantly influencing the molding of SMC powders. Owing to its remarkable insulation properties, the Si added to SMC powder minimized eddy current loss and enhances electromagnetic performance. However, an excessive increase in Si content can compromise mechanical strength and formability. This phenomenon occurs as silicon content increases in the Fe-Si alloy, increasing brittleness and potentially resulting in internal cracks during the molding process, which impedes the development of a compact structure at elevated Si concentrations. To address this limitation, this study selected Si contents of 5.0 wt.% and 6.5 wt.% for experimentation. Although 6.5 wt.% Si is recognized for its exceptional electromagnetic properties, its limited formability requires careful adjustments to the production process. Conversely, 5.0 wt.% Si exhibits relatively good formability, but it potentially degrades electromagnetic properties. Therefore, an objective of this study is to comparatively analyze these two compositions to derive an optimal balance.

To address the reduced formability caused by elevated silicon content, hot compaction was conducted at temperatures exceeding 300 °C as opposed to standard room-temperature forming. In addition to facilitating the formation of a dense microstructure by inducing plastic deformation between powder particles, hot compaction can potentially enhance mechanical strength by suppressing internal microcrack formation. However, hot compaction can also induce undesirable phase transformations and excessive energy consumption, necessitating the determination of an optimal forming temperature. Therefore, primary forming temperatures of 25 °C (room temperature), 300 °C, 400 °C, and 500 °C were selected as temperature points.

Subsequently, annealing was applied at 720 °C for 10 min as a standard post-forming process to relieve residual stresses generated during compaction and to stabilize the interparticle insulation layer. Annealing was considered a binary process variable “Yes” or “No”.

In contrast to the conventional single-stage pressing method (green compact), a two-stage pressing process (secondary compaction) was incorporated to optimize the final product density and minimize porosity. This secondary compaction process was also conducted at elevated temperatures, with secondary forming temperatures of 25 °C, 300 °C, 400 °C, 500 °C, 600 °C, and 625 °C selected as temperature points. In the initial primary forming stage, the powder was pressed to produce a basic shape (green bulk), then subjected to annealing to alleviate internal stresses and improve interparticle bonding strength, which was essential for preventing crack formation during the final forming process.

A full factorial design, comprising 96 unique parameter combinations derived from these factors, was generated, and these conditions are summarized in Table 8. Experiments were repeated under identical conditions to ensure data reliability, and the formability, density, and electromagnetic properties were compared and analyzed for each condition.

Toroidal specimens were fabricated using a hydraulic press with a maximum capacity of 100 tons, with a compaction pressure of 8 tons per square unit. These specimens, prepared according to the design of experiment (DOE) conditions detailed in Table 8, were then subjected to a fixed-condition heat treatment to finalize their properties. The heat treatment was conducted in a mixed hydrogen and argon gas atmosphere. Given the approximately 6–8 times higher thermal conductivity of hydrogen compared to argon, this mixed gas atmosphere facilitated uniform heat distribution, enhancing the efficiency of the heat treatment heat treatment process and minimizing thermal deformation. Furthermore, the potent reducing power of hydrogen facilitates the reduction of metal oxides to their metallic states, thereby improving surface cleanliness. Regarding Fe powder sintering, iron oxide (FeO) is reduced to Fe, enabling the fabrication of high-density sintered products with densities exceeding 92%. To prevent excessive decarburization, while leveraging the benefits of hydrogen, the hydrogen concentration was limited to 10% within the predominantly argon atmosphere; consequently, a gas mixture of 10% hydrogen and 90% argon was selected [41,42].

The heat treatment was performed at 700 °C, a temperature known to effectively alleviate internal residual stress and minimize internal defects in SMCs, thereby optimizing their magnetic properties. However, excessive temperatures above 700 °C can induce phase transformations and resistance changes in the internal coating layer, potentially degrading magnetic performance. Therefore, considering the stabilization of the internal structure and the balance of magnetic characteristics, 700 °C was ultimately selected as the heat treatment temperature [43,44,45].

To more effectively evaluate the characteristics of the SMC powder, a toroidal core geometry was selected as the evaluation model due to its provision of a uniform magnetic field distribution, its advantage in assessing magnetic properties, and its resemblance to actual motor cores, enhancing the reliability of the experimental results. The outer and inner diameters of the core are 20.3∅ and 12.7∅, respectively. The core height, influenced by the target powder filling amount of 8 g, was controlled to be between 5 and 6 mm. For each experimental condition, three independently fabricated toroidal core specimens were prepared and evaluated to ensure reproducibility and the statistical significance of the measured properties. The detailed experimental factors and their corresponding levels are systematically summarized in Table 9, outlining the DOE applied in this study. The experimental results obtained using this described DOE array are summarized in Table S1.

#### 2.2.3. Measurement Method

This study focused on evaluating the magnetic properties of SMC cores, with particular emphasis on density, permeability, and core loss performance. The height, outer diameter, and inner diameter of the toroidal cores were measured using a micrometer, and the weight of each specimen was determined using an electronic balance. Accordingly, the effective magnetic path length, $l_{e}$, for each specimen was calculated using Equation (1).

To determine the permeability of the toroidal cores, reactance measurements were performed using a Precision LCR meter (4284A, Agilent Technologies, Santa Clara, CA, USA) across a frequency range of 100–10,000 Hz. These measurements were conducted with a 10-turn coil wound around each toroidal core.

(1) $$l_{e}=\frac{\pi (D_{outer}-D_{inner})}{\ln(D_{outer}/D_{inner})}$$

(2) $$\mu_{r}=\frac{L\times l_{e}}{\mu_{0}\times N^{2}\times A}$$

where L, $l_{e}$, $\mu_{0}$, N, and A denote the inductance, effective magnetic path length, permeability of free space, number of coil turns, and effective cross-sectional area of the core, respectively.

Core losses generally comprise hysteresis loss, eddy current loss, and residual loss. To qualitatively assess these losses, the quality factor (Q-factor) was measured, enabling the qualitative evaluation of the trade-off between electromagnetic performance and losses. The Q-factor is prevalent as an indicator to minimize losses and maximize efficiency in magnetic components, as defined in Equation (3):

(3) $$Q=\frac{1}{\tan\delta }=\frac{\omega L}{R}$$

where ω, L, and R represent the angular frequency, inductance, and equivalent resistance, respectively. A higher Q-factor indicates a material with lower losses and higher efficiency.

## 3. Powder Compaction Simulation

This study attempts to derive optimal conditions based on experimental analysis, involving data measurement via the actual testing of 96 parameter combinations. Subsequently, the two samples exhibiting the most superior characteristics are selected for simulation, establishing a method for digitalizing and streamlining future experimental process. An objective of this study is to maximize experimental efficiency and enhance the reliability of deriving optimal design parameters. Based on the 96 combinations, the two samples exhibiting the most remarkable characteristics are chosen for simulation. Conditions with the highest density are prioritized for selection, and simulations are performed based on these. This approach facilitates the computerization and simplification of subsequent experimental procedures, and reproducibility can be secured by verifying the significant correlation between the results of actual experiments and simulations. Consequently, future research can apply a wider array of factor combinations by reducing the number of experiments and minimizing experimental costs and time.

### 3.1. Yield Criteria for Porous Materials

Considering the influence of porosity on the deformation behavior during powder compaction, the Shima–Oyane constitutive model was employed (4) [46,47]. This model enables the quantitative evaluation of the porosity effect on mechanical properties and allows for a more accurate prediction of stress distribution under local density gradients.

(4) $$\sigma_{eq}=\sigma_{y}(1-Df)$$

where $\sigma_{y},\;D,\;and\;f$, denote the yield strength of the material, a material constant, and porosity, respectively. This equation directly explains how an increase in the porosity of a material reduces its yield stress.

### 3.2. Simulation Condition

**Figure 4:**
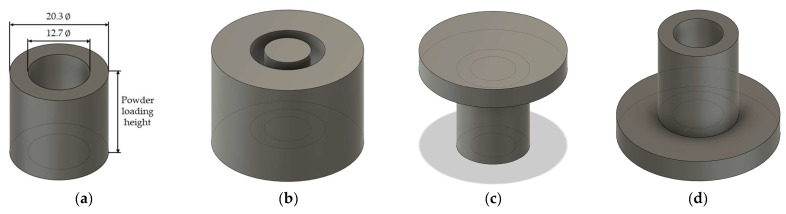
FEA simulation model feature: (**a**) workpiece, (**b**) die, (**c**) upper punch, and (**d**) lower punch.

The simulation model illustrating the experiment is presented in Figure 4. Given that the press employed in the experiment is a uniaxial compression type, the simulation utilizes a model in which the lower punch is fixed relative to the die. The loading conditions applied to the upper punch functioned as the termination criteria for the simulation. The press utilized in the experiment applies to a load of 8 tons per unit area, which was converted to 78,453.2 N per unit area for the simulation. The powder loading height was determined by considering the apparent density of the original powder and converting it to relative density.

A constant friction coefficient of 0.2 was applied between the powder and die wall, according to literature reports on ferrous powder systems undergoing uniaxial compaction. This value is within the experimentally validated range (μ = 0.2–0.3) and helps simulate realistic pressure transmission and densification behavior [48,49].

## 4. Results

### 4.1. Experimental Results

The experimental data, summarizing four experimental parameters and three resultant characteristics, are presented in Supplemental Table S1. The selected resultant characteristics are the density, permeability, and Q-factor. Considering the operating frequency of the vehicle motor, the permeability and Q-factor were evaluated at three frequencies: 0.1, 1.0, and 10 kHz.

The statistical results are summarized in Table 10. Data loss occurred in 9 out of 96 experiments under the 6.5 wt.% silicon content condition, primarily due to missing values resulting from molding defects. This data loss in the Fe-Si SMC with high silicon content is attributed to the impact of high silicon content on mechanical formability and electromagnetic properties, with molding defects being a primary cause. Therefore, to ensure data reliability and prevent analytical errors caused by missing values, rows containing incomplete data were excluded from the analysis.

**Figure 5:**
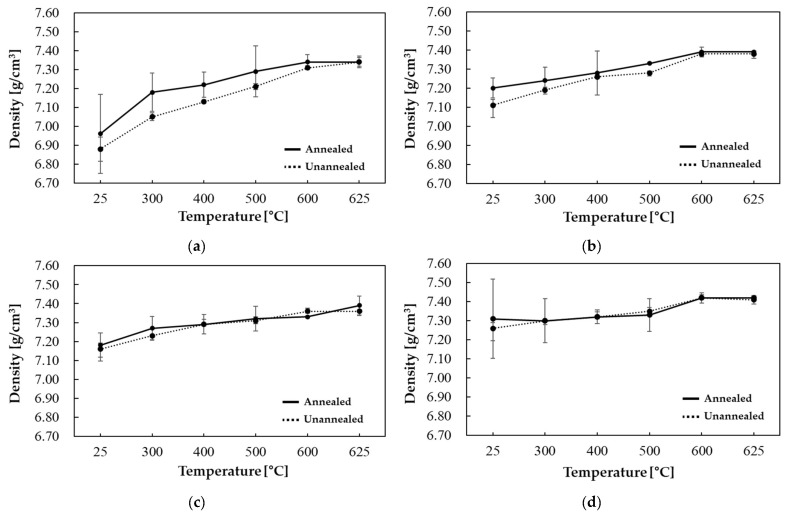
Fe-5.0 wt.%Si density results: 1st forming temperature (**a**) 25 °C, (**b**) 300 °C, (**c**) 400 °C, and (**d**) 500 °C.

Figure 5 illustrates the density results of samples containing 5.0 wt.% silicon, obtained under varying primary forming temperatures. Error bars represent the standard deviation of three repeated measurements. Generally, density levels increase with increasing primary and secondary forming temperatures, and the impact of annealing diminishes as the primary forming temperature rises. This is attributed to the partial stress relief and enhanced interparticle diffusion occurring during forming at elevated temperatures, resulting in a comparatively smaller reduction in residual stress from subsequent annealing and thus less density enhancement. The maximum density is observed at a primary forming temperature of 500 °C, followed by annealing and a secondary forming temperature of 625 °C.

**Figure 6:**
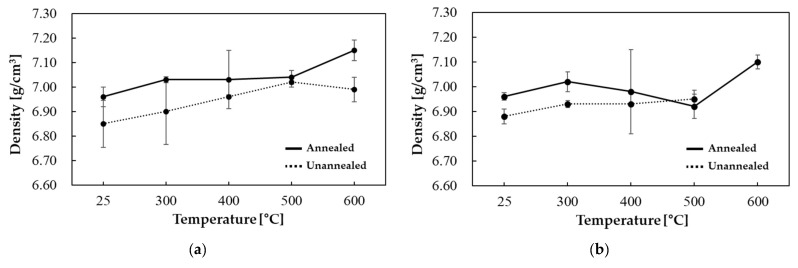

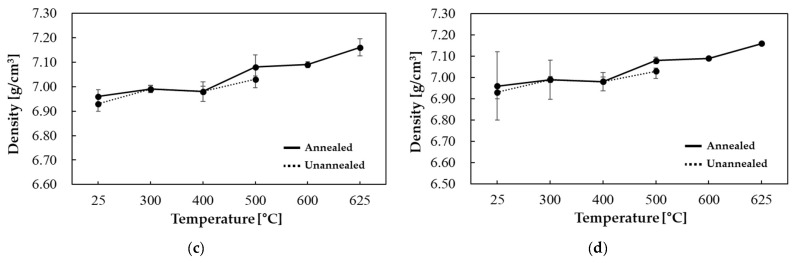
Fe-6.5 wt.%Si density results: 1st forming temperature (**a**) 25 °C, (**b**) 300 °C, (**c**) 400 °C, and (**d**) 500 °C.

Figure 6 illustrates the density results for a silicon content of 6.5 wt.% as a function of the primary forming temperature. For a 6.5 wt.% Si content, a significant number of missing values were observed at secondary forming temperatures above 600 °C. This is attributable to the decrease in interatomic bonding strength resulting from the introduction of silicon into iron, which results in an increase in the material’s brittleness. Specifically, at a high silicon content of 6.5 wt.%, the material’s ductility is significantly diminished, and the risk of crack formation due to stress concentration increases [50,51].

**Figure 7:**
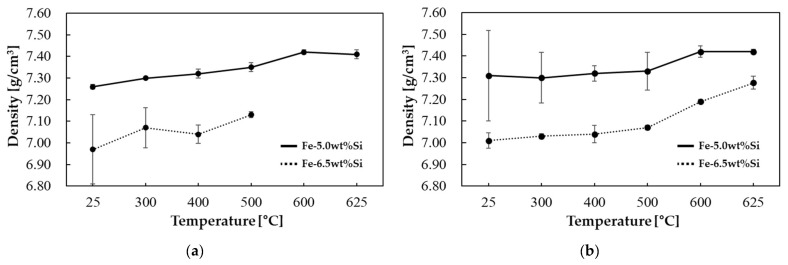
Density results according to silicon content at a primary compaction temperature of 500 °C: (**a**) unannealed and (**b**) annealed.

Following primary forming at 500 °C and subsequent annealing, density exhibited a decreasing trend with increasing silicon content, as illustrated in Figure 7. Furthermore, the influence of powder content control remained unaffected by the annealing process.

**Figure 8:**
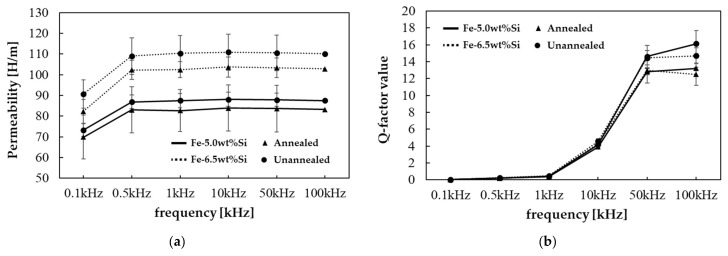
Permeability and Q-factor results according to silicon content and annealing process: (**a**) permeability and (**b**) Q-factor.

The permeability and Q-factor of Fe-Si powder were analyzed using a toroidal core, and the obtained results are presented in Figure 8. The analysis was conducted under primary and secondary molding temperatures of 500 °C. Regarding permeability, the 6.5 wt.% Si powder generally exhibited better performance than the 5.0 wt.% Si powder, suggesting a significant influence of Si content on magnetic property enhancement, independent of density variations attributable to the molding conditions. Furthermore, unannealed specimens demonstrated higher permeability than annealed specimens, likely due to insulation coating degradation during annealing leading to increased magnetic losses. The permeability trend relative to frequency exhibited a relatively low permeability at low frequencies, gradually increasing towards higher frequencies, attributed to the high electrical resistivity of Fe-Si material suppressing eddy current losses at high frequencies. The Q-factor, which indicates core loss performance, exhibited similar performance up to 10 kHz. Beyond this point, the 5.0 wt.% Si powder showed higher values, with the unannealed specimens demonstrating superior performance. In addition, the Q-value tended to increase with increasing frequency, confirming the excellent core loss reduction characteristics of Fe-Si powder in high-frequency applications.

This study fabricated Fe-Si-based SMCs using a process-focused approach involving dual-stage compaction and annealing optimization without polymer insulation or orientation treatment. Under these conditions, the specimens achieved densities of up to 7.42 g/cm^3^ and permeability values of 90–100 at 1 kHz.

Huang et al. fabricated Fe-based SMCs via warm compaction and reported a density of 6.38 g/cm^3^ and a permeability of 45.8 measured at 100 kHz. In comparison, this study achieved higher densities of 7.28–7.42 g/cm^3^, corresponding to a 14.1–16.3% increase, and a permeability range of 70–110 under the same frequency, thus indicating an improvement of 52.8–140.2%. Song et al. employed PTFE-based insulation and optimized thermal treatment to reduce core losses, reporting a permeability of 56 at 50 kHz. Under matching conditions, our specimens exhibited permeability values of 70–95, indicating a 25.0–69.6% improvement while maintaining comparable core loss characteristics.

These comparisons indicate that high magnetic performance and densification can be simultaneously achieved via mechanical process control alone, offering a practical and scalable route for high-frequency SMC applications.

### 4.2. PCC Analysis

The collected experimental data were analyzed for linear correlations between factors using the PCC analysis, as expressed in Equation (5) [52,53].

(5) $$\rho_{X,Y}=\frac{E[\left(X-\mu_{X}\right)\left(Y-\mu_{Y}\right)]}{\sigma_{X}\sigma_{Y}}$$

where E represents the expected value, $\mu_{X}$ and $\mu_{Y}$ denote the means of the respective variables, and $\sigma_{X}$ and $\sigma_{Y}\;$ denote their corresponding standard deviations.

**Figure 9:**
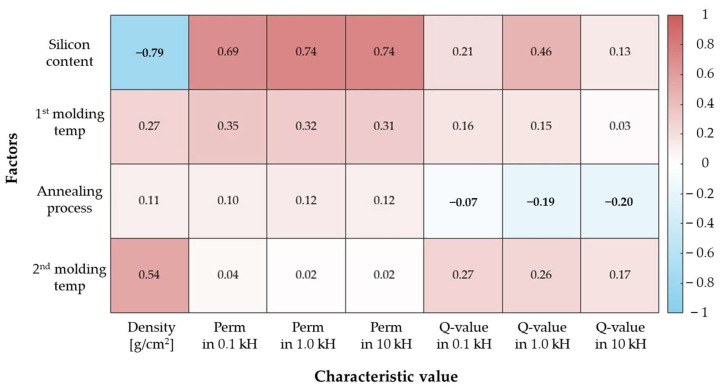
PCC analysis results.

The influence of molding process parameters, including Si content, primary molding temperature, annealing treatment, and secondary molding temperature, on density and electromagnetic properties (permeability and Q-factor) was investigated via a PCC analysis. The obtained results are presented as a heatmap in Figure 9. The analysis demonstrated that increasing Si content reduced density; however, an increase in permeability, with a partial improvement in the Q-factor, reduced iron loss. This suggests that although the addition of Si is effective in enhancing magnetic properties, it also increases brittleness, thereby reducing density. Although the primary molding temperature exhibited a weak positive correlation with density, it did not significantly impact the permeability or Q-factor. Annealing treatment generally exhibited low or negative correlations with all properties; in particular, a weak negative correlation with Q-factor suggested that annealing might even induce an increase in iron loss. The weak negative correlation between annealing and Q-factor is attributable to the thermal degradation of the phosphate-PI insulation layer. During the annealing process, elevated temperatures may induce partial decomposition or structural relaxation of the polymer-based coating, which diminishes its dielectric strength and reduces interparticle resistance. This effect increases eddy current pathways within the compacted core, increasing core losses and decreasing the Q-factor [54]. Finally, secondary molding temperature exhibited a strong positive correlation with density enhancement. Although it did not significantly impact permeability, it exerted a minor positive effect on reducing iron loss. Considering these analyses holistically, optimizing silicon content and secondary molding temperature to achieve a balance between density and magnetic properties is crucial for manufacturing SMCs. Furthermore, the application of annealing treatment requires careful consideration, as its impact on magnetic properties is limited or potentially detrimental.

### 4.3. Simulation Result

The simulation identified the conditions that yielded the maximum density for each silicon content experiment: a primary molding temperature of 500 °C, followed by annealing and then a secondary molding temperature of 625 °C. Prototypes fabricated under these conditions exhibited a density of 7.42 g/cm^3^ at a silicon content of 5.0 wt.% and 7.28 g/cm^3^ at 6.5 wt.%. The initial conditions for the simulation were established based on the apparent density of the raw powder for each silicon content, as presented in Table 11.

**Figure 10:**
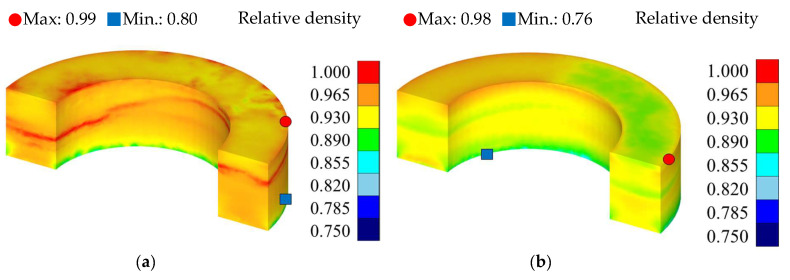
Relative density results: (**a**) Fe-5.0 wt.%Si and (**b**) Fe-6.5 wt.%Si.

**Figure 11:**
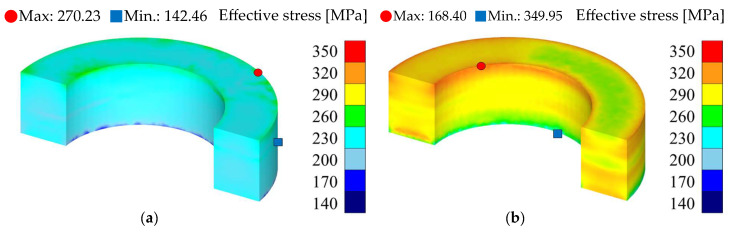
Effective stress results: (**a**) Fe-5.0 wt.%Si and (**b**) Fe-6.5 wt.%Si.

**Figure 12:**
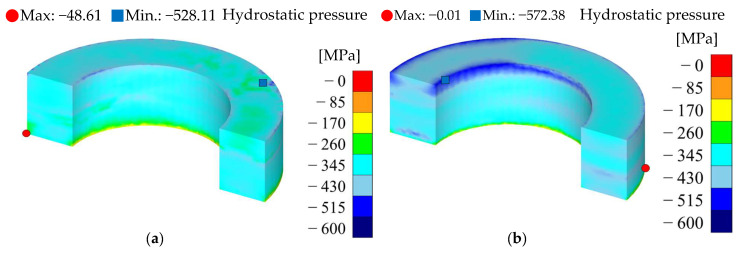
Hydrostatic pressure results: (**a**) Fe-5.0 wt.%Si and (**b**) Fe-6.5 wt.%Si.

The density, effective stress, and hydrostatic pressure results for each silicon content condition are presented in Table 12 and Figure 10, Figure 11 and Figure 12. The analysis results were visualized in a half-core geometry to elucidate the internal state. The maximum and minimum values for each property were also presented to compare the property distribution.

Figure 10 presents the density simulation results for each silicon content condition. Under the 5.0 wt.% silicon content condition, the average density was approximately 7.41 g/cm^3^, exhibiting a 0.13% error compared to the prototype measurement of 7.42 g/cm^3^. Conversely, under the 6.5 wt.% condition, the average density was approximately 7.25 g/cm^3^, with an error of 0.41% compared to the prototype value of 7.28 g/cm^3^. In both cases, the simulation results indicated a tendency for the maximum and minimum densities to be located at the top and bottom of the core, respectively. This is interpreted as a density distribution non-uniformity phenomenon, frequently observed in uniaxial pressing, which can result in reduced powder flowability and an increased risk of defects during actual molding.

Furthermore, while the average density was higher under the 5.0 wt.% condition, the maximum–minimum density deviation was more evident under the 6.5 wt.% condition, which is attributable to the increased brittleness and decreased plastic deformation capability of the powder resulting from the increased silicon content, resulting in reduced internal compression uniformity. Under the 5.0 wt.% condition, the average density was high, exceeding 7.4 g/cm^3^, with a strong density concentration observed in the central region of the product. Hence, further evaluation of potential insulation layer damage and powder coating layer fracture in this region is crucial via the microstructural analysis of the actual prototype.

The effective stress results for each silicon content condition corroborate the previously analyzed density results, as presented in Figure 11. Generally, a certain level of yield stress is required for powder to undergo plastic deformation. Powders with higher silicon content tend to exhibit increased brittleness and resistance to plastic deformation. Certainly, the 6.5 wt.% condition exhibits an overall high level of effective stress distribution, with an average effective stress approximately 36% higher than that observed in the 5.0 wt.% condition. This contributes to the incomplete densification and lower density formation of the 6.5 wt.% powder under the same compaction pressure conditions.

Furthermore, both conditions exhibit a tendency for maximum stress and maximum density points to form at the top of the core, while minimum stress and minimum density points form at the bottom. This is attributed to the uneven pressure transmission inherent in uniaxial pressing, which is a primary cause of density distribution imbalance. Consequently, the effective stress analysis quantitatively elucidates the influence of silicon content in Fe-Si powder on formability and densification, accurately reflecting the correlation with the density results.

Figure 12 illustrates the hydrostatic stress distribution for each silicon content condition. Hydrostatic stress, representing the average isotropic compressive stress within the powder during compaction, influences interparticle contact and densification, functioning as an essential indicator for assessing powder compatibility. Generally, higher hydrostatic stress facilitates enhanced interparticle bonding, resulting in higher densities; however, it also proportionally increases the internal reaction forces acting on the die and punch.

According to the simulation results, the hydrostatic stress at 6.5 wt.% (−396.99 MPa) was approximately 11.5% higher than at 5.0 wt.% (−356.13 MPa). This suggests that an increased silicon content enhances resistance to plastic deformation, resulting in a larger accumulation of compressive stress accumulation within the powder under the same external load. However, despite the high hydrostatic stress at 6.5 wt.%, the increased brittleness of the powder impedes sufficient plastic flow, resulting in a lower overall average density. Notably, such elevated hydrostatic stress can escalate side pressure and reaction forces on the die walls and punch during compaction, thereby increasing the risk of die failure and punch wear. Under high-pressure conditions, fatigue accumulation or localized stress concentration within the die material can drastically reduce lifespan or cause dimensional deviations during repeated compaction cycles. Therefore, predictive analysis and reinforcement designs regarding hydrostatic stress and die stress distribution, in addition to compaction pressure, are deemed essential when compacting powders with high silicon content.

A hydrostatic stress analysis was employed to quantitatively assess the molding efficiency and stress concentration behavior as a function of silicon content. Notably, the 6.5 wt.% Si powder exhibited low density, even under high pressure, owing to increased resistance to plastic deformation and heightened brittleness.

### 4.4. Comparison Between Simulation and Bulk Prototype

#### 4.4.1. Bulk Morphology Analysis of Fe-Si

**Figure 13:**
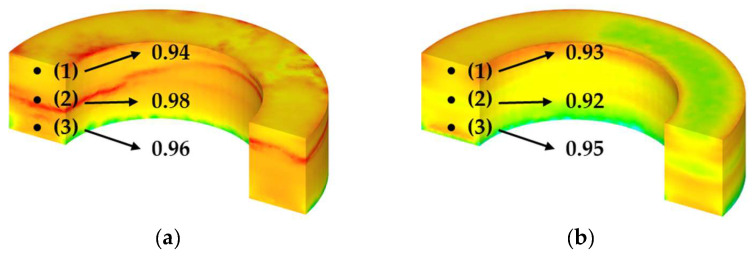
The relative density at three height points of the toroidal core: Si content (**a**) 5.0 wt.%; (**b**) 6.5 wt.%; and (**1**) top, (**2**) middle, and (**3**) bottom regions.

The reliability of density distribution and its correlation with the actual forming structure characteristics were analyzed by comparing the simulation results with a toroidal bulk prototype. As illustrated in Figure 13, the cross-section of the toroidal specimen was segmented into three distinct regions: top (0.5 mm height), middle (3.0 mm height), and bottom (4.5 mm height). The evaluation specifically addressed the density distribution and corresponding microstructural differences across these top, middle, and bottom regions. Furthermore, the structural integrity of the insulation layer was examined utilizing a combination of SEM and focused ion beam (FIB)-TEM analyses.

The relative density measurements of the prototypes, presented in Figure 13, revealed the following density distributions for the 5.0 wt.% Si specimen: 0.94 (top), 0.98 (middle), and 0.96 (bottom). In contrast, the 6.5 wt.% Si specimen exhibited densities of 0.93 (top), 0.92 (middle), and 0.95 (bottom) at the same locations. The 5.0 wt.% specimen showed the highest density in the middle region, whereas the 6.5 wt.% specimen exhibited a relatively higher density in the bottom region. This difference is attributed to the varying flowability and deformation resistance of the powder depending on the silicon content during uniaxial pressing with the upper punch. Specifically, the increased brittleness of the 6.5 wt.% Si powder appears to have hindered compression transfer to the center, resulting in a concentration of compressive stress in the bottom region.

**Figure 14:**
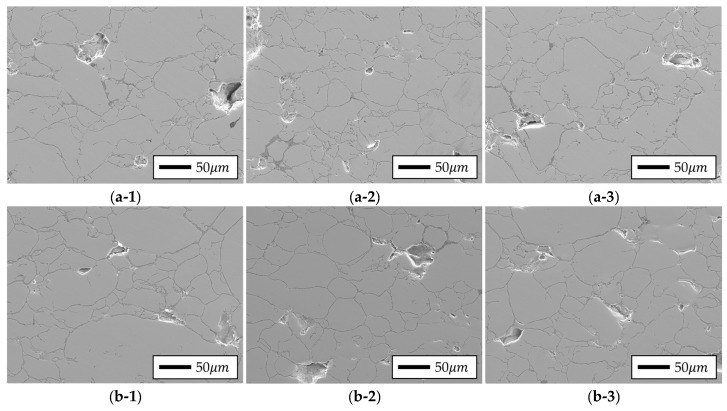
Toroidal core SEM image results: (**a-1**–**a-3**) 5.0 wt.% Si content in top, middle, and bottom regions; (**b-1**–**b-3**) 6.5 wt.% Si content in top, middle, and bottom regions.

As illustrated in Figure 14, the SEM analysis corroborated these density distribution trends with corresponding microstructural characteristics. In the 5.0 wt.% Si specimen, the middle region exhibited the fewest and smallest pores, with a slight increase in porosity in the bottom region and a relatively high pore concentration in the top region. This result directly agrees with the measured relative densities, suggesting that enhanced densification in the middle region led to improved interparticle bonding. Conversely, the 6.5 wt.% Si specimen exhibited the fewest pores in the bottom region and the most in the middle, which is attributed to insufficient compression of the central region due to the high resistance to plastic deformation and low flowability of the 6.5 wt.% Si powder.

The upper section of the 5.0 wt.% Si specimen exhibited numerous large particles exceeding 150 μm, along with significant necking and localized deterioration of the interparticle insulation coating, interpreted because of excessive pressure concentration in the top region, resulting in particle rearrangement and coating damage. In contrast, necking phenomena were observed in both the top and bottom regions of the 6.5 wt.% Si specimen, with a significant necking structure evident in the bottom region due to the strong interparticle contact pressure. These microstructural analyses empirically support the impact of silicon content and location-specific compression conditions on the interparticle bonding mechanisms and insulation layer stability.

#### 4.4.2. Localized Chemistry at Interfaces of Bulk Prototype

**Figure 15:**
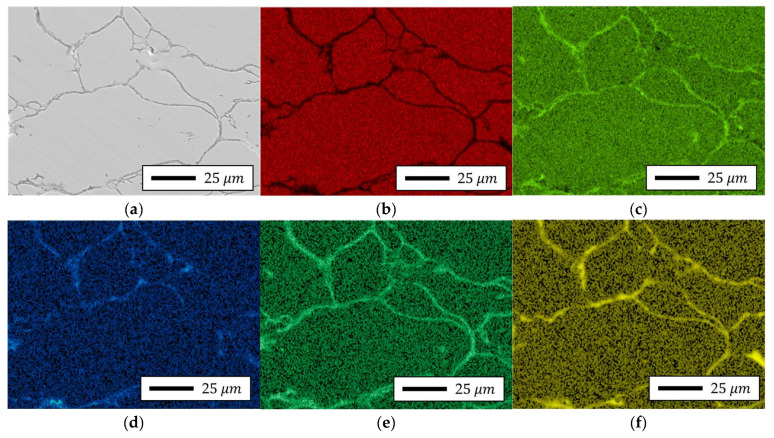
SEM-EDS image results of Fe-5.0 wt.%Si bulk specimen: (**a**) SEM-EDS image site map, (**b**) Fe, (**c**) Si, (**d**) P, (**e**) O, (**f**) Mo.

**Figure 16:**
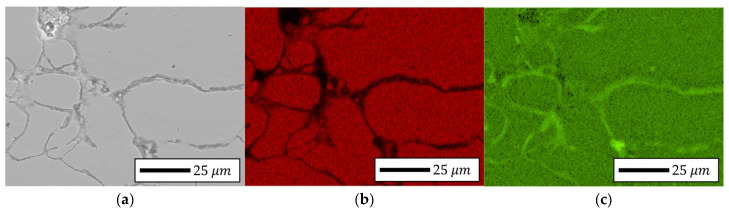

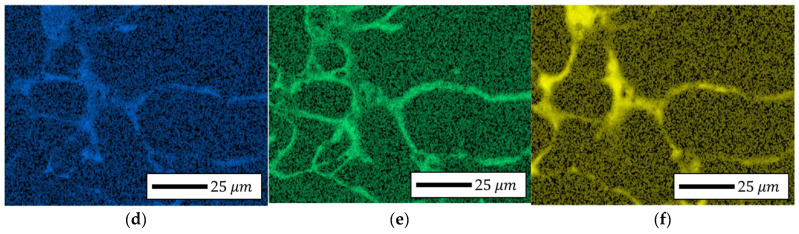
SEM-EDS image results of Fe-6.5 wt.%Si bulk specimen: (**a**) Fe, (**b**) Si, (**c**) P, (**d**) O, (**e**) Mo, and (**f**) S.

To investigate the localized distribution of insulating components, an energy-dispersive X-ray spectroscopy (EDS) analysis was conducted on selected interparticle regions within SEM images of both the 5.0 wt.% and 6.5 wt.% Si specimens. The results, as illustrated in Figure 15 and Figure 16, revealed a relatively uniform interfacial composition in the 5.0 wt.% samples, whereas the 6.5 wt.% specimens exhibited significant enrichment of silicon, phosphorus, and oxygen at particle boundaries, indicating the formation of a chemically enriched insulation layer. To further assess the structural and compositional characteristics of this layer, a transmission electron microscopy (TEM) analysis was performed on FIB-prepared cross-sections of both compositions. The 5.0 wt.% samples exhibited thin, uniform interfacial regions with minimal contrast, while the 6.5 wt.% specimens exhibited a thicker, partially amorphous, and chemically heterogeneous structure.

**Figure 17:**
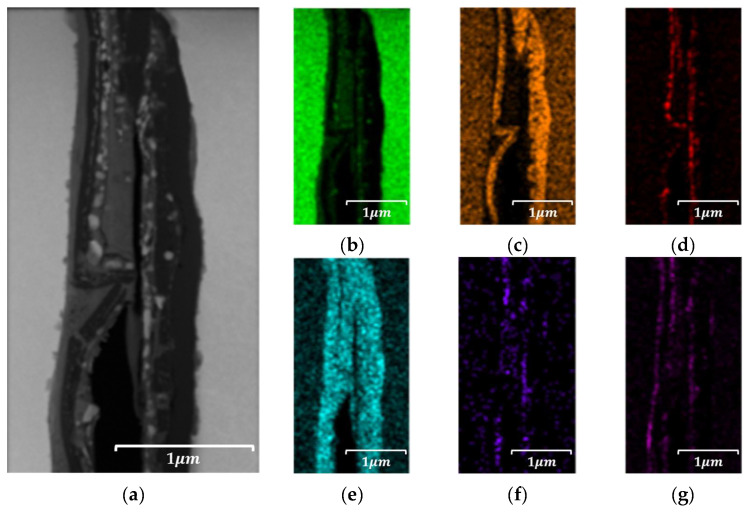
TEM-EDS results of Fe-5.0 wt.%Si bulk specimen: (**a**) TEM image site map, (**b**) Fe, (**c**) Si, (**d**) P, (**e**) O, (**f**) Mo, and (**g**) S.

**Figure 18:**
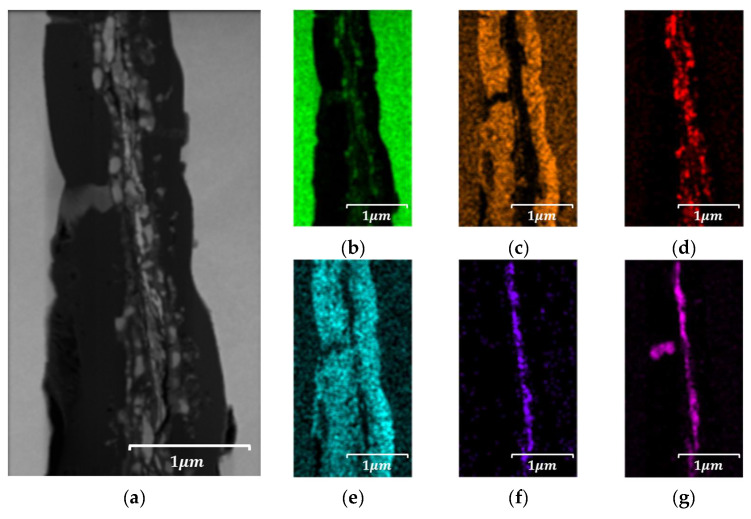
TEM-EDS results of Fe-6.5 wt.%Si bulk specimen TEM—EDS results: (**a**) TEM image site map, (**b**) Fe, (**c**) Si, (**d**) P, (**e**) O, (**f**) Mo, and (**g**) S.

In the 5.0 wt.% specimen, an approximately 1 μm wide transition layer was observed at the interparticle interface, as illustrated in Figure 17. Within this region, a thin insulating film, tentatively identified as a phosphate-based compound, was distributed around the particle periphery with a thickness of approximately 10 nm or less. Conversely, the 6.5 wt.% specimen exhibited a transition region of approximately 1.2 μm, With the phosphate film observed to be approximately 400 nm thick, as illustrated in Figure 18. This difference is attributable to variations in the film’s composition and growth mechanism resulting from the increased Si content, suggesting that higher Si content induces structural changes in the interparticle interface characteristics along with a thicker coating layer. Magnetic property measurements revealed that an average Q-factor value at 10 kHz for the 5.0 wt.% Si specimens of 3.94 (standard deviation: 1.12) for the 5.0 wt.% Si specimens and a slightly improved average Q-factor of 4.10 (standard deviation: 0.52) for the 6.5 wt.% Si specimens. These results suggest that the thicker insulation layer contributed to enhanced eddy current suppression and improved consistency in high-frequency magnetic performance. However, the structural non-uniformity observed in the 6.5 wt.% specimens may still pose a risk for localized dielectric breakdown under high-frequency excitation or prolonged use conditions. Accordingly, the correlation between the insulation layer morphology and magnetic performance was qualitatively interpreted and discussed.

The obtained findings indicate that in addition to magnetic properties, increasing silicon content also affects various other aspects, including the molding density distribution, interparticle bonding, and stability of the insulating coating. Specifically, density concentration in the upper region and necking phenomenon may include structural damage due to excessive molding pressure. This suggests the necessity of molding pressure adjustments and uniform powder distribution design to address these challenges.

#### 4.4.3. Crystal Structures of Bulk Prototype

**Figure 19:**
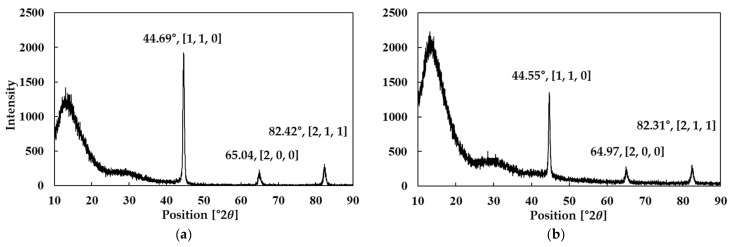
XRD results of bulk toroidal core: Si content (**a**) 5.0 wt.% and (**b**) 6.5 wt.%.

Furthermore, an XRD analysis of bulk specimens was conducted to verify the crystal structure of the prototypes. As illustrated in Figure 19, the primary diffraction peaks (Bragg peaks) for both the 5.0 wt.% and 6.5 wt.% Si conditions agreed with the reference pattern for Fe_1.6_Si_0.4_, consistent with the XRD analysis of the powder form. All diffraction peaks corresponded to a cubic crystal system, indicating the stability of the crystal structure throughout heat treatment and molding processes. These findings confirm that the Fe-Si alloy maintains the same phase in both powder and bulk states, with no undesirable phase transformations or structural deformations occurring during high-temperature molding and heat treatment.

## 5. Conclusions

This study investigated the optimization of process parameters to enhance the formability and electromagnetic properties of Fe-Si-based SMCs. Two silicon content conditions, 5.0 wt.% and 6.5 wt.%, were selected as target compositions, and their comprehensive effects on formability, mechanical structural stability, and magnetic properties were evaluated. Specifically, the density, effective stress, and hydrostatic pressure distribution during the powder compaction process were comparatively analyzed via simulations and experimental measurements. Furthermore, the internal structure and insulation layer stability were quantitatively examined via a microstructure analysis at various locations. Considering the growing demand for next-generation vehicle applications, including artificial intelligence (AI)-driven autonomous systems, this study also provides foundational insights into the applicability of Fe-Si-based SMCs in smart motor designs.

The influence of molding parameter control was systematically investigated via the implementation of a full factorial experimental design encompassing 96 condition combinations. A PCC analysis was subsequently employed to quantitatively determine the correlations between key molding process parameters and material properties. The findings revealed a strong negative correlation between silicon content and density; however, a strong positive correlation was observed between silicon content and permeability. This suggests that increasing silicon content enhances magnetic properties but simultaneously increases powder brittleness and reduces plastic flowability, thereby impairing formability and highlighting a trade-off relationship. Furthermore, the secondary forming temperature was identified as the most influential factor for achieving high density, while annealing treatment negatively affected core loss, potentially reducing the Q-factor.

Unlike previous studies that focused separately on either mechanical or magnetic properties, this study systematically addressed the trade-off between formability and electromagnetic performance by applying a full-factorial experimental design, PCC analysis, and finite element simulation. Furthermore, this study provides novel quantitative insights into insulation layer degradation mechanisms at elevated silicon contents, which have not been comprehensively reported before. These findings support both advancement in experimental methodology and a deeper understanding of material behavior, functioning as fundamental data to support the practical application of Fe-Si SMCs as next-generation high-speed, high-efficiency motor core materials. Such material-based optimization studies are expected to contribute to advancing AI-assisted design and control platforms for future automotive systems. Ultimately, this study is expected to provide a novel reference for the precise design of SMCs with remarkable magnetic properties in the low-frequency range, establishing a technical foundation for the high-reliability design and commercialization of Fe-Si-based SMCs. Future research will build upon these findings, focusing on interparticle resistance analysis, annealing condition optimization, residual stress validation, and advanced powder processing to mitigate the inherent trade-offs between formability and magnetic properties.

## Figures and Tables

**Table 1:** Thermal conductivity of Fe-5.0 wt.%Si.

Temperature (°C)	Thermal Diffusivity (mm^2^/s)	Specific Heat (J/gK)	Thermal Conductivity (W/mK)
25	5.38	0.45	17.7
100	5.56	0.47	19.0
200	5.66	0.52	21.4
300	5.58	0.54	21.9
400	5.33	0.57	22.2
500	4.91	0.58	20.8
600	4.61	0.63	21.0
700	3.81	0.70	19.4
800	2.79	0.74	15.0
900	4.03	0.77	22.7

**Table 2:** Thermal conductivity of Fe-6.5 wt.%Si.

Temperature (°C)	Thermal Diffusivity (mm^2^/s)	Specific Heat (J/gK)	Thermal Conductivity (W/mK)
25	4.20	0.45	14.2
100	4.34	0.54	17.6
200	4.50	0.57	19.0
300	4.59	0.58	20.0
400	4.60	0.60	20.6
500	4.51	0.64	21.6
600	4.21	0.91	28.6
700	3.76	1.07	30.2
800	4.17	0.97	30.4
900	4.51	0.93	31.3

**Table 3:** Thermal expansion coefficients of Fe-5.0 wt.%Si and Fe-6.5 wt.%Si.

Temperature (°C)	Coefficient of Thermal Expansion (1e-6/K)	
	Fe-5.0 wt.%Si	Fe-6.5 wt.%Si
100	11.25	11.27
200	11.94	11.90
300	12.50	12.61
400	12.91	13.07
500	13.25	13.54
600	13.58	14.18
700	13.79	14.68
800	13.97	15.03
900	14.24	15.32

**Table 4:** Fe-5.0 wt.%Si raw powder chemical composition.

Element	Fe	Si	O
wt.%	94.85	5.02	0.13

**Table 5:** Fe-6.5 wt.%Si raw powder chemical composition.

Element	Fe	Si	O
wt.%	93.62	6.54	0.16

**Table 6:** Particle size of Fe-5.0 wt.%Si and Fe-6.5 wt.%Si raw powders.

Si Content	5.0 wt.%	6.5 wt.%
average particle size of powder	79.136	99.804

**Table 7:** Coating composition of Fe-Si powder.

Composition	H_3_PO_4_	PI	MoS_2_
Wt.%	1.0	0.5	1.0

**Table 8:** Design of control factors and level.

Factor	Level 1	Level 2	Level 3	Level 4	Level 5	Level 6
Si content [wt.%]	5.0	6.5				
1st forming temperature [°C]	RT	300	400	500		
2nd forming temperature [°C]	RT	300	400	500	600	625
annealing process	Yes	No				

**Table 9:** Experimental parameter set.

Experimental Parameter	Value/Condition
forming pressure	8 tons per square unit
1st forming temperature	RT, 300 °C, 400 °C, 500 °C
2nd forming temperature	RT, 300 °C, 400 °C, 500 °C, 600 °C, 625 °C
annealing temperature	700 °C
annealing atmosphere	10% H_2_ + 90% Ar
specimen shape	toroidal core
target specimen weight	8 g
number of specimens per condition	3 specimens
insulation coating composition	1.0 wt.% H_3_PO_4_ + 0.5 wt.% PI + 1.0 wt.%MoS_2_

**Table 10:** Summary of experimental results.

Index	Density [g/ cm^3^]	Permeability in 0.1 kH	Permeability in 1.0 kH	Permeability in 10 kH	Q-Factor in 0.1 kH	Q-Factor in 1.0 kH	Q-Factor in 10 kH
Count	87	87	87	87	87	87	87
Min	6.85	3.26	3.95	3.99	0.02	0.27	2.18
Max	7.42	99.92	125.15	126.33	0.04	0.51	7.18

**Table 11:** Boundary conditions of simulation.

Silicon Content	$\mathrm{Apparent}\;\mathrm{Density}\;[g/{cm}^{3}]$	Initial Relative Density	Powder Loading Height [mm]
5.0 wt.%	3.27	0.419	12.009
6.5 wt.%	3.08	0.395	12.302

**Table 12:** Comparison of optimal case results according to DOE.

Silicon Content	Average Relative Density	Average Effective Stress [MPa]	Average Hydrostatic Pressure [MPa]
5.0 wt.%	0.95	220.25	−356.13
6.5 wt.%	0.93	301.12	−396.99

## Data Availability

The original contributions presented in the study are included in the article; further inquiries can be directed to the corresponding author.

## Supplementary Materials

The following supporting information can be downloaded at https://www.mdpi.com/article/10.3390/ma18102321/s1, Table S1: experimental data.
